# Influence of Pelvic Tilt on Polyethylene Wear after Total Hip Arthroplasty

**DOI:** 10.1155/2015/327217

**Published:** 2015-07-16

**Authors:** Taro Tezuka, Yutaka Inaba, Naomi Kobayashi, Hiroyuki Ike, So Kubota, Masaki Kawamura, Tomoyuki Saito

**Affiliations:** Department of Orthopaedic Surgery, Yokohama City University, Yokohama 236-004, Japan

## Abstract

We aimed to evaluate the effects of pelvic tilt on polyethylene wear after total hip arthroplasty (THA). A total of 105 joints treated with primary THA were included; conventional polyethylene (CPE) liners were used in 43 hips and highly cross-linked polyethylene (HXLPE) liners were used in the remaining 62 hips. The pelvis was tilted 6° posteriorly in the standing position as compared to the supine position, which resulted in significant increases of 1.7° and 2.8° in cup inclination in the CPE and HXLPE groups, respectively. Moreover, the change in pelvic tilt resulted in significant increases of 3.6° and 4.9° in cup anteversion in the CPE and HXLPE groups, respectively. For the CPE group, multiple regression analysis showed a significant association between the angle of pelvic tilt (PTA) and cup inclination and the polyethylene wear ratio. The adjusted *R* ^2^ of the regression model was larger for measures obtained in the standing position as compared to the supine position. For the HXLPE group, there was no significant relationship between radiographic parameters and polyethylene wear. Close observation of polyethylene wear is recommended for patients with severe posterior pelvic tilt who have undergone THA with conventional polyethylene.

## 1. Introduction

Inclination and anteversion of the cup in total hip arthroplasty (THA) affect clinical outcomes, including postoperative hip joint range of motion, risk of postoperative dislocation, and increased polyethylene wear, which can lead to osteolysis around the implant and cause loosening [1–3].

An increase in the posterior tilt position of the pelvis after THA has recently been reported [4, 5]. A number of studies have investigated the relationship between pelvic tilt and the angle of the cup of the THA [6–8]. However, the specific relationship between pelvic tilt and polyethylene wear has not been investigated. Therefore, our study addressed two specific goals. The first was to quantify pelvic tilt after THA in the supine and standing positions and to evaluate the relationship of postural change in pelvic tilt with the angle of the cup. The second was to use multiple regression analysis to evaluate the relationship of pelvic tilt and the angle of the cup with polyethylene wear of the THA.

## 2. Materials and Methods

This study was a retrospective evaluation of patients who underwent primary THA at a single center, between 1986 and 2006. The methods and procedures were approved by the authors' institutional review board. Outcomes of a total of 105 joints, from 96 patients, were evaluated. Conventional polyethylene liners were used for 43 hips in 34 patients (CPE group), and highly cross-linked polyethylene liners were used in the other 62 hips from 62 patients (HXLPE group). The average age at surgery was 59 ± 6.7 years in the CPE group and 64 ± 8.7 years in the HXLPE group; there were no significant differences in age between the groups (*p* = 0.56, unpaired Student's *t*-test). The average follow-up period was 16.4 ± 3.4 years for the CPE group and 5.7 ± 2.2 years for the HXLPE group (*p* < 0.01; unpaired Student's *t*-test).

The porous coated anatomic THA system (Stryker Howmedica Osteonics Corp., Allendale, NJ), with a high-density polyethylene liner (1050 GUR sterilized with gamma radiation in air) and a 26 mm femoral head component, was used for all hips in the CPE group. For the HXLPE group, the Secure Fit stem, Super Secure Fit stem, or CentPiller stem systems (Stryker Orthopaedics, Mahwah, NJ, USA) were used for 46 hips, and the VerSys fiber metal midcoat stem system (Zimmer International, Warsaw, IN, USA) was used for 16 hips. Forty-six Trident AD cups (Stryker Orthopaedics) and 16 Trilogy cups (Zimmer International) were used as cups, and 46 Crossfire liners (1050 GUR, which was irradiated by 10 Mrad radiation and annealed polyethylene, Stryker Orthopaedics) and 16 Longevity liners (1050 GUR, which was 10 Mrad electron beam-irradiated and melted, Zimmer International) were used as polyethylene liners. A 26 mm femoral head was used in all cases.

For all cases, anterior-posterior (AP) pelvic radiographs were obtained in the supine and standing positions at the final follow-up session. The angle of inclination and degree of anteversion of the cup and the pelvic tilt angle (PTA) were measured from both supine and standing position radiographs. Polyethylene wear was also measured from the radiographs in the supine position. Anteversion of the cup was measured using the methods of Lewinnek [1]. The minor axis of the cup was designated as *D*_1_ and the major axis was designated as *D*_2_ on AP pelvic radiographs, and the angle of anteversion (*α*) was calculated as *α* = sin^−1^⁡*D*_1_/*D*_2_ + 5°. The PTA was calculated using the method of Doiguchi [5, 9]. The lateral diameter (*T*) and longitudinal diameter (*L*) of the pelvic cavity were measured on AP pelvic radiographs, and the PTA was calculated as −67 × *L*/*T* + 55.7° for men and −69 × *L*/*T* + 61.6° for women. According to this method, the larger the PTA, the more the pelvis tilts posteriorly, with a mean PTA of 20° reported for healthy females. Linear polyethylene wear was measured using Roentgen Monographic Analysis (Roman) version 1.70 (Institute of Orthopaedics, Oswestry, UK) [10], following the method of Livermore et al. [11]. With the Roman software, 8 points on the edge of the cup were chosen and averaged to generate an edge and center. This process was repeated for the femoral head, and the displacement vector between the centers of the femoral head and cup components was measured. To rule out deformation of the polyethylene liner immediately after surgery, the difference in the central position of the femoral head between the 1-year postoperative and the final follow-up time points was measured [12]. The polyethylene wear rate after 1 year was determined by fitting a regression line to the data points at 1 year and at the final follow-up. The annual rate of wear (mm/year) was quantified as the slope of the regression line.

The change in PTA measured in the supine and standing positions was defined as ΔPTA; the change in the inclination and anteversion of the cup was defined, in a similar manner, as Δinclination and Δanteversion, respectively. Simple regression analysis was performed to analyze the relationships between the ΔPTA and Δinclination and between ΔPTA and Δanteversion in both THA groups. Additionally, Pearson's correlation coefficients were calculated to evaluate the association between the polyethylene wear rate and radiographic measurements after THA. Independent determinants of the polyethylene wear rate were identified by multiple linear regression. The radiographic measurements were entered simultaneously. The adjusted coefficient of multiple determination (adjusted *R* ^2^) was used to indicate the extent to which the variability in the polyethylene wear rate was accounted for by the independent variables. Standardized regression coefficients (*β*) and associated *p* values were determined to assess statistical significance (*p* < 0.05). All statistical analyses were performed using SPSS version 21.0 (IBM Corp., Armonk, NY, USA).

## 3. Results

**Figure 1:**
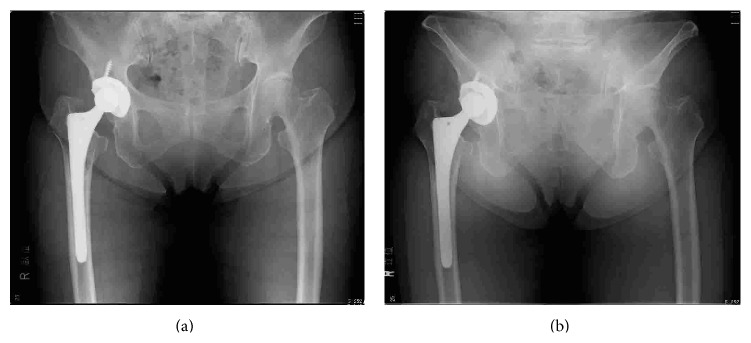
Radiographs of a 72-year-old woman treated with right hip THA. An HXLPE liner was implanted, and the annual polyethylene wear rate was 0.008 mm/year. (a) AP pelvic radiograph in the supine position 5 years after THA; cup inclination was 46°, cup anteversion was 16°, and PTA was 31°. (b) AP pelvic radiograph in the standing position shows that the pelvis is tilted posteriorly. The PTA was 59°, leading to an increase in cup inclination and anteversion; moreover, cup inclination was 52° and cup anteversion was 38°.

In the CPE group, the PTA was 24.7 ± 7.5° in the supine position and 31.6 ± 9.7° in the standing position. From the supine to standing positions, the cup inclination changed from 40.2 ± 6.7° to 41.9 ± 7.1° (*p* < 0.01), and cup anteversion changed from 14.6 ± 5.5° to 18.2 ± 6.3° (*p* < 0.01). The PTA in the HXLPE group in the standing and supine positions were similar to those of the CPE group, with measures of 24.0 ± 8.3° and 30.3 ± 10.2°, respectively. There was a significant 6.3° posterior tilt of the pelvis in the standing position as compared to that in the supine position (Figure 1). From the supine to standing positions, the cup inclination increased from 41.6 ± 6.6° to 44.4 ± 6.5°, and cup anteversion increased from 20.0 ± 8.8° to 24.9 ± 9.7°, indicating a significant increase in the values from the standing position to the supine position (*p* < 0.01, *p* < 0.01) (Table 1).

**Figure 2:**
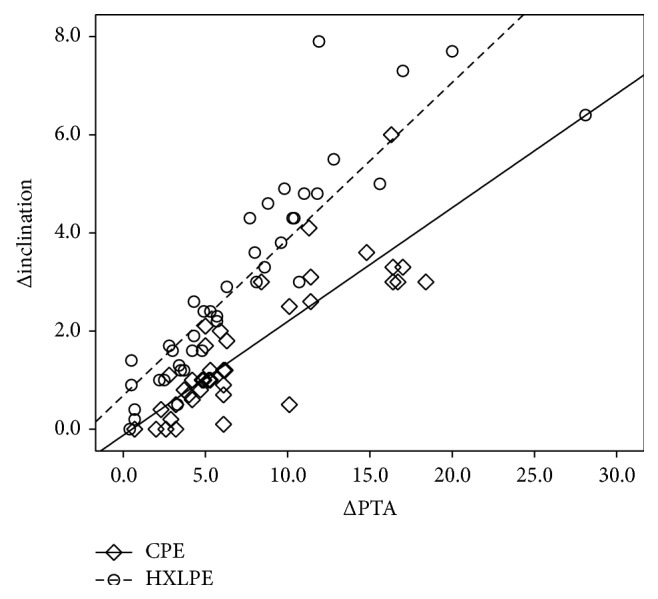
Simple linear regression between ΔPTA and Δinclination. In both groups, there was a positive correlation between ΔPTA and Δinclination (CPE group: *r* = 0.67; *p* < 0.01; HXLPE group: *r* = 0.43; *p* = 0.02). For every 10° of pelvis tilt, the cup inclination increased by 3°.

Figure 2 shows the results of the simple regression analysis between ΔPTA and Δinclination for the CPE and HXLPE groups. In both groups, there was a positive correlation between ΔPTA and Δinclination (CPE group: *r* = 0.67, *p* < 0.01; HXLPE group: *r* = 0.43, *p* = 0.02). Based on the gradient of the graph, a 10° posterior tilt of the pelvis was associated with an increase in inclination of 3°.

**Figure 3:**
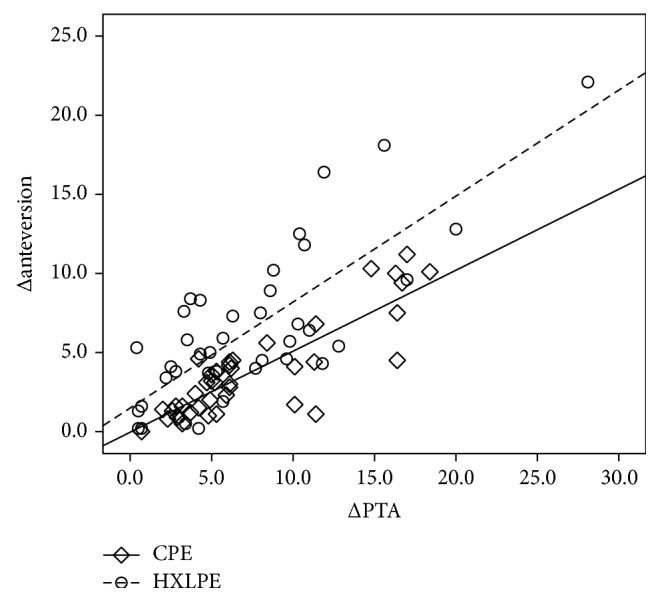
Simple linear regression between ΔPTA and Δanteversion. In both groups, there was a positive correlation between ΔPTA and Δanteversion (CPE group: *r* = 0.74; *p* < 0.01; HXLPE group: *r* = 0.82; *p* < 0.01). For every 10° of pelvis tilt, the cup anteversion increased by 7°.

Figure 3 shows the results of simple regression analysis between ΔPTA and Δanteversion for the CPE and HXLPE groups. In both groups, there was a positive correlation between ΔPTA and Δanteversion (CPE group: *r* = 0.74, *p* < 0.01; HXLPE group: *r* = 0.82, *p* < 0.01). Based on the gradient of the graph, a 10° posterior tilt of the pelvis was associated with an increase in anteversion of 7°.

**Figure 4:**
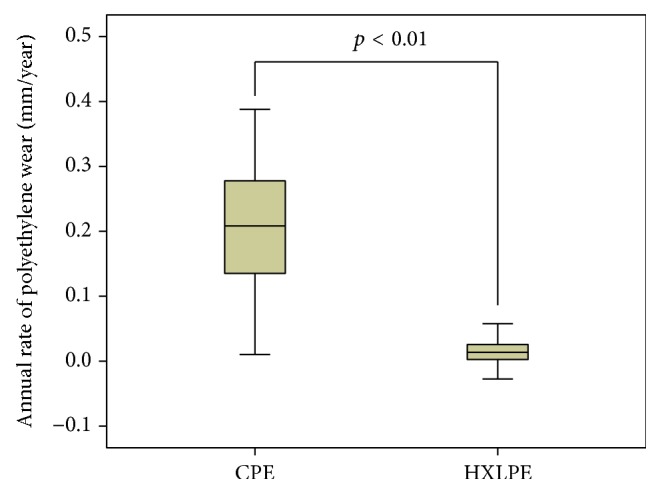
The annual polyethylene rate after THA. The polyethylene annual wear rate was 0.21 ± 0.10 mm/year for the CPE group and 0.014 ± 0.05 mm/year for the HXLPE group; there was a significant difference in this value between the groups (unpaired Student's *t*-test).

The rate of polyethylene wear was 0.21 ± 0.11 mm/year in the CPE group and 0.014 ± 0.05 mm/year in the HXLPE group, and there was a significant difference in this value between the groups (*p* < 0.01; unpaired Student's *t*-test) (Figure 4).

Pearson's correlation coefficients between the rate of polyethylene wear and radiographic parameters in the supine and standing positions are reported in Table 2. For the CPE group, PTA in the standing position and cup inclination and anteversion in the supine and standing positions significantly influenced the rate of polyethylene wear. However, there was no significant relationship between polyethylene wear rate and these radiographic parameters for the HXLPE group. There was no significant correlation between ΔPTA and annual rate of polyethylene wear in both groups.

For the CPE group, multiple regression analysis showed that the PTA (standard regression coefficient [*β*] = 0.292; *p* = 0.04) and cup inclination angle (*β* = 0.398,  *p* < 0.01) were significantly associated with the polyethylene wear rate in the supine position. The final model accounted for 9% (adjusted *R* ^2^ = 0.09) of the variance in polyethylene wear rate. In the standing position, as in the supine position, multiple regression analysis showed that the PTA (*β* = 0.388; *p* = 0.03) and cup inclination angle (*β* = 0.417, *p* < 0.01) were significantly associated with the polyethylene wear rate and the final model accounted for 16% (adjusted *R* ^2^ = 0.16) of the variance in the polyethylene wear rate (Table 3). For the HXLPE group, there was no significant correlation between the annual polyethylene linear wear rate and radiographic parameters (Table 4).

## 4. Discussion

It is well known that polyethylene linear wear is one of the causes of aseptic loosening leading to THA revision. Several factors that affect polyethylene wear have been reported, such as the size of the femoral head; the thickness of the polyethylene liner [13]; patient age [14], BMI [15], and postoperative activities of daily living [13, 14]; position of the cup [16]; and cup inclination and offset [17]. To our knowledge, our study is the first to assess the association between polyethylene wear and pelvic tilt.

Patil et al. [18] evaluated the contact stress of the hip joint by finite-element analysis and reported a concomitant increase in contact stress on the joint surface with the increase in the angle of inclination of the cup. Conversely, contact stress decreased as the angle of anteversion of the cup increased posterior tilting of the pelvis increased both the inclination and anteversion of the cup. Therefore, we were mainly concerned about the manner in which pelvic tilt influences polyethylene wear.

From the results of our current study, both inclination and anteversion of the cup increased through the posterior tilt of the pelvis after THA. Cup inclination and anteversion increased by 3° and 7°, respectively, with a 10° posterior tilt of the pelvis. Lembeck et al. [19] reported that each degree of pelvic tilt requires a 0.7° correction in the angle of anteversion of the acetabular cup. Babisch et al. [20] also reported that cup inclination changed by approximately 0.3° and cup anteversion changed by approximately 0.8° per 1° change of pelvic tilt. Our clinical results were consistent with the results of these modeling studies and, therefore, were considered to be appropriate.

For the CPE group, the annual polyethylene liner wear rate correlated with cup inclination and pelvic tilt angle. Cup anteversion did not correlate with polyethylene wear rate and, therefore, was not considered to be a major factor influencing polyethylene wear. Multivariate regression analysis in the present study indicated that pelvic tilt and cup inclination were significant factors for polyethylene wear, and the current study is the first to report the influence of pelvic tilt on polyethylene wear after THA. The adjusted *R* ^2^ values for pelvic tilt and cup inclination were larger in the standing position than in the supine position; hence, the radiographic parameters in the standing position are better indicators of the influence of polyethylene wear as compared to those in the supine position.

For the HXLPE group, although inclination and anteversion increased through an increase in posterior tilt of the pelvis after THA, we could not find any significant correlation between polyethylene annual linear wear rate and these parameters. We believe that this is largely due to the improvement of the quality of polyethylene liner. However, considering the results from modeling studies described above, contact stress might increase as a function of increased cup inclination in cases with severe pelvic tilt after THA in this group as well.

Another concern was whether the change in pelvic tilt from supine to standing position (i.e., ΔPTA) influences polyethylene wear. We identified some cases where the amount of change in pelvic tilt from the supine to standing positions was large. Initially, we thought that such large changes in pelvic tilt might be one of the factors influencing polyethylene wear; however, we did not find the amount of postural change in pelvic tilt to be a significant factor of the annual rate of polyethylene wear.

The limitations of this study should be considered. First, due to the retrospective nature of this study, the follow-up period for the HXLPE group was significantly shorter than for the CPE group. Dai et al., who followed up patients with THA for a period of 131 ± 10 months, reported that the migration of the femoral head at an average period of 3.4 months after THA accounted for 56% of the total migration of the femoral component in the first 2 years and that the degree of wear in the first postoperative year accounted for nearly 40% of the total wear [12]. Dai et al. determined that after an early change in polyethylene, known as the creep response, the rate of wear decreased gradually with time and stabilized. In our study, we excluded the measures of wear over the first year after THA and compared the polyethylene wear over steady periods, with an average follow-up period of the HXLPE group of 5.7 years. Therefore, we do not believe that the difference in the follow-up period between the CPE and HXLPE groups influenced our results.

In our study, we also did not evaluate the change in pelvic tilt before and after THA, as preoperative pelvic radiographs in the supine and standing positions had not been obtained in a sufficient number of patients before THA. Therefore, the effect of the change of pelvic tilt after THA on polyethylene wear is unknown. Our results do show, however, that pelvic tilt at any time point after THA could be a factor influencing polyethylene wear. Close observation is recommended for patients with severe posterior pelvic tilt measures who have undergone THA using conventional polyethylene.

## 5. Conclusion

Compared to the supine position, there was a significant increase in posterior pelvic tilt in the standing position, resulting in increased cup inclination and anteversion. In THA with conventional polyethylene liners, pelvic tilt in the supine and standing positions correlated with the annual rate of polyethylene linear wear as well as with cup inclination. In HXLPE hips, pelvic tilt had no effect on the annual rate of wear of the liner.

## Figures and Tables

**Table 1:** Pelvic tilt angle (PTA) and the angle of inclination and anteversion of the cup in the supine and standing positions.

	CPE group	*p *	HXLPE group	*p *
PTA (°)				
Supine	24.7 ± 7.5	<0.01	24.0 ± 8.3	<0.01
Standing	31.6 ± 9.7		30.3 ± 10.2	
Cup inclination (°)				
Supine	40.2 ± 6.7	<0.01	41.6 ± 6.6	<0.01
Standing	41.9 ± 7.1		44.4 ± 6.5	
Cup anteversion (°)				
Supine	14.6 ± 5.5	<0.01	20.0 ± 8.8	<0.01
Standing	18.2 ± 6.3		24.9 ± 9.7	

Unpaired Student's *t*-test.

**Table 2:** Pearson's correlation coefficients between polyethylene wear rate and factors of pelvic tilt angle (PTA) and the angle of inclination and anteversion of the cup.

Parameter	Position	CPE group		HXLPE group	
		*r*	*p*	*r*	*p*
PTA	Supine	0.24	0.11	0.21	0.06
	Standing	0.34	0.02	0.1	0.36

Cup inclination	Supine	0.39	<0.01	0.27	0.16
	Standing	0.43	<0.01	0.07	0.53

Cup anteversion	Supine	0.23	0.02	0.18	0.1
	Standing	0.37	0.02	0.09	0.93

ΔPTA		0.29	0.06	0.18	0.38

**Table 3:** Multiple regression analysis between conventional polyethylene wear rate and factors of pelvic tilt angle (PTA) and the angle of inclination and anteversion of the cup.

Position	Parameter	Adjusted *R* ^2^	Regression coefficient	Standard regression coefficient (*β*)	*p *
Supine	PTA	0.09	0.004	0.292	0.04
	Cup inclination		0.005	0.398	0.01
	Cup anteversion		0.02	0.03	0.54

Standing	PTA	0.16	0.002	0.388	0.03
	Cup inclination		0.002	0.417	<0.01
	Cup anteversion		0.03	0.085	0.59

**Table 4:** Multiple regression analysis between highly cross-linked polyethylene wear rate and factors of pelvic tilt angle (PTA) and the angle of inclination and anteversion of the cup.

Position	Adjusted *R* ^2^	Regression coefficient	Standard regression coefficient	*p *
Supine				
PTA	0.07	−0.003	−0.58	0.06
Cup inclination		0.003	0.35	0.07
Cup anteversion		−0.004	−0.48	0.17

Standing				
PTA	0.03	−0.001	−0.1	0.42
Cup inclination		0.001	0.06	0.63
Cup anteversion		−0.001	−0.01	0.94
